# Healing through Histidine: Bioinspired Pathways to Self-Healing Polymers via Imidazole–Metal Coordination

**DOI:** 10.3390/biomimetics4010020

**Published:** 2019-02-27

**Authors:** Stefan Zechel, Martin D. Hager, Tobias Priemel, Matthew J. Harrington

**Affiliations:** 1 Laboratory for Organic and Macromolecular Chemistry (IOMC), Friedrich Schiller University Jena, Humboldtstr. 10, 07743 Jena, Germany; stefan.zechel@uni-jena.de; 2 Jena Center for Soft Matter (JCSM), Friedrich Schiller University Jena, Philosophenweg 7, 07743 Jena, Germany; 3 Department of Chemistry, McGill University, 801 Sherbrooke Street West, Montreal, QC H3A 0B8, Canada; tobias.priemel@mail.mcgill.ca

**Keywords:** self-healing, histidine, imidazole, metal coordination, mussel byssus, hydrogels, metallopolymers

## Abstract

Biology offers a valuable inspiration toward the development of self-healing engineering composites and polymers. In particular, chemical level design principles extracted from proteinaceous biopolymers, especially the mussel byssus, provide inspiration for design of autonomous and intrinsic healing in synthetic polymers. The mussel byssus is an acellular tissue comprised of extremely tough protein-based fibers, produced by mussels to secure attachment on rocky surfaces. Threads exhibit self-healing response following an apparent plastic yield event, recovering initial material properties in a time-dependent fashion. Recent biochemical analysis of the structure–function relationships defining this response reveal a key role of sacrificial cross-links based on metal coordination bonds between Zn^2+^ ions and histidine amino acid residues. Inspired by this example, many research groups have developed self-healing polymeric materials based on histidine (imidazole)–metal chemistry. In this review, we provide a detailed overview of the current understanding of the self-healing mechanism in byssal threads, and an overview of the current state of the art in histidine- and imidazole-based synthetic polymers.

## 1. Introduction: The Role of Histidine–Metal Coordination in Self-Healing Behaviors of Biopolymeric Materials

### 1.1. Brief Introduction to Self-Healing Materials

The functional lifetime and efficacy of materials is limited by the onset of damage, whether they are composites comprising aircraft and automobiles, concretes comprising bridges and buildings or polymeric materials comprising device components. Damage can take the form of catastrophic cracks and fractures, nonreversible plastic deformation or gradual fatigue and failure via formation of microcracks in the material structure, among other forms [1]. Regardless of the exact manner of the damage, efforts to prevent and alleviate the effects of material damage are a massive preoccupation for material scientists and engineers, as the real-world consequences of material damage and failure can be disastrous (e.g., metal fatigue in aerospace engineering). A typical strategy is to overcompensate by overdesigning a device so that the materials comprising it are not overtaxed by the typical physical loading of the material. However, this increases the manufacturing costs and necessitates vigilant maintenance, requiring many man hours or added features. More recently, engineering of smart and stimuli-responsive properties into materials, including self-healing response, has begun to provide another route for mitigating the deleterious effects of materials damage [1,2].

Self-healing describes the capacity of a material to actively repair damage, and it is becoming a critical design feature with the potential for extending the functional lifetime of polymers, composites, metals and concretes and for allowing materials to function near their theoretical optimum since they no longer need to be overdesigned [1,2,3]. As such, enormous efforts over the last 20 years have aimed at engineering this highly desirable property into a wide range of material classes through various strategies and at multiple hierarchical length scales. Of course, not all damage is created equally. As already mentioned, material damage ranges from fatigue microcracking, plastic yield, and outright fracture, which all require different sorts of healing response [1]. For the relevant background in this regard, we direct the reader to many excellent reviews that provide essential background on the range of damage and healing encountered in both living and engineered systems [1,2,3,4].

Regardless of the sort of damage being addressed, self-healing is typically categorized into several different classifications depending on the spontaneity of the healing process and whether or not healing requires external chemical additives. Autonomous healing describes a process that occurs spontaneously once the damage occurs (i.e., damage is the trigger for healing), whereas nonautonomous healing requires an external input to stimulate the healing response (e.g., temperature, electrical current, magnetic fields, and light exposure) [2]. Intrinsic self-healing describes a process, which is inherent to the material composition and structure itself and does not require external additives to initiate healing, whereas extrinsic healing does require an external chemical agent (outside of the material itself) to initiate healing at the damage site [5]. Thus, the ideal self-healing material would be a combination of autonomous and intrinsic healing that proceeds spontaneously, actively, and dynamically upon damage application. However, it is challenging to achieve this inherent dynamicity and maintain material performance relevant for real-world applications. In light of these challenges, researchers have looked to nature for material design inspiration based on the capacity of living organisms and natural materials to heal damage.

### 1.2. Biological Role Models for Self-Healing Materials

Rational engineering of self-healing polymers, concretes, and composites came of age only in the early 2000s [6]. However, nature has evolved a wide variety of self-healing responses over eons through natural selection and thus, provides a useful role model for the development and design of new engineered material through biomimicry and bioinspiration [4]. Table 1 provides an overview of well-characterized self-healing responses in living systems. Self-healing of tissues in living organisms is an obvious evolutionary advantage in terms of survival to reproductive age. Life would be very different if we were obliged to live permanently with childhood bumps, bruises, cuts, and broken bones. Luckily, our bodies are capable of major feats of regeneration and reengineering as displayed in wound repair and bone mending. In these examples, the cells within our tissues act as tiny factories, which are tasked with rebuilding tissues [7,8]. The extraordinary chain of biochemical signaling events responsible for cellular-driven healing are reviewed in detail elsewhere [7]. However, the general distilled concepts underlying the response (i.e., temporary, local mobility) rather than the chemical level details of biological wound healing has been successfully exploited toward the development of highly effective self-healing composites [9]. The very first example of this was the development by White and co-workers of self-healing epoxy resins containing encapsulated healing agents that were activated as a crack propagated through embedded capsules [6]. The liquid precursor flowed into the arrested crack and was subsequently polymerized with the aid of catalysts integrated into the epoxy structure. This basic concept was later developed into more complex vascular healing resembling the network of arteries in the circulatory system [10]. While these examples of autonomic, but extrinsic self-healing composites represent clear achievements of human engineering, they mimic only the spirit of biological healing and do not capture the chemical complexities involved. In fact, the inherent complexity of the natural wound healing or bone mending is currently well beyond what chemists and engineers can hope to replicate at the bench [4,7]. However, in simpler biological self-healing materials and biopolymers, understanding the underlying chemistry of healing can be extremely useful toward the development of self-healing materials. Indeed, nature also offers excellent examples of acellular healing in biopolymeric materials, usually comprised of proteins, in which healing is both autonomous and intrinsic and arises from specific chemical level details of the protein sequence, structure, and cross-linking (Table 1) [4]. Because the healing response can be distilled down to chemical and physical level features of the proteins, it offers a much simpler path for bioinspired design. By far, the most prominent example of bioinspired self-healing from an acellular biopolymeric source is the use of metal coordination cross-linking as a dynamic and reversible sacrificial bond, which breaks and reforms on time scales relevant to the material function [4,11].

### 1.3. Metal Coordination as a Dynamic Cross-Linking Strategy in Natural Materials

Metal coordination complexes are dynamic molecular interactions that have an enormous range of functions that blur the line between biology and synthetic chemistry. For inorganic chemists, synthetic metal complexes have a large diversity of different properties and play important roles in topics like (photo)catalysis [16] and optical properties [17,18]. On the other hand, protein–metal coordination is familiar to most biologists and biochemists due to the integral physiological roles that metal ions, such as Fe, Zn, Cu, and Mn play in the active sites of enzymes, gas transport molecules, photosystems, and electron transport chains [19,20]. Typically, the active sites of such proteins consist of a metal ion coordinated with specific, highly conserved amino acid residues, and it is the redox properties of the metal ion that are critical to the desired function [11,19,20]. However, more recently, it has been discovered that a number of biological materials utilize protein–metal interactions in load-bearing role, functioning as mechanical cross-links that have crucial effect due to their dynamic properties [11].

Metal coordination interactions are intriguing as load-bearing cross-links due to their intermediate status between pure covalent and noncovalent interactions. Protein–metal interactions are typically mediated by specific amino acid residues including histidine, aspartic acid, glutamic acid, and cysteine and post-translationally modified amino acids such as 3,4-dihydroxyphenylalanine (DOPA) and phosphoserine [11,19,20]. These amino acid ligands donate a lone pair of electrons into hybrid orbitals in the outer shell of the metal ions. Such protein–metal interactions possess bond strengths that are much larger than typical noncovalent interactions such as hydrogen bonding and van der Waals; yet, they still possess fast bond kinetics, making them much more labile than covalent bonds [11,21,22,23]. Furthermore, the bonding interaction and geometry are highly specific to the amino acid ligands present and the preferred coordination geometry of the metal ion utilized [19,20]. These characteristics make metal coordination bonds highly useful as dynamic supramolecular bonds, and biological organisms, through the mechanism of natural selection, have evolved to employ these interactions for important mechanical functions. For a thorough examination of the range of different metal coordination interactions found in biological materials and the specific roles they play in functional material properties, we refer the reader to a recent review article [11]. In particular, it is worth mentioning the key role that DOPA–metal coordination has been shown to play in the mechanical performance of biological materials, which has been utilized toward the design and fabrication of bioinspired adhesives, coatings and self-healing polymers. However, this has been well reviewed previously [24].

### 1.4. Histidine as a Versatile Ligand for Tuning Mechanical Performance of Biopolymers

**Figure 1:**
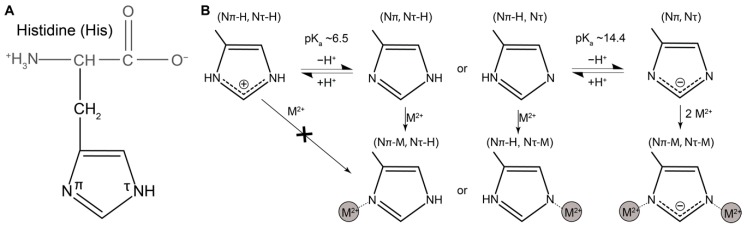
Histidine chemistry and metal binding. (**A**) Chemical structure of the amino acid histidine (His) under physiological conditions (pH ≈ 7.5). (**B**) Depending on pH and other solution conditions, the histidine imidazole side chain can exist in a number of forms. Metal (M^2+^) binding can only occur when a proton is lost from at least one nitrogen atom.

Of the 20 naturally occurring amino acids, histidine stands out as one of the most versatile in terms of possible inter- and intramolecular interactions [25]. The side group of histidine is an imidazole ring with two nitrogen atoms that are labeled N_π_ and N_τ_ (although other nomenclatures exist) [25,26] (Figure 1A). Histidine has a first p*K*_a_ value of ≈6.5 that falls within the physiological range. As such, it can undergo protonation/deprotonation of one of the nitrogen atoms, such that the side chain is fully protonated and positively charged under acidic conditions and under neutral/basic condition, one of the nitrogen atoms (usually the N_π_) is deprotonated and the side chain is uncharged (Figure 1B) [25,26]. The second nitrogen generally has a much higher p*K*_a_ of >14, and when lost, the side chain takes the negatively charged form (Figure 1B). Depending on the conditions, the histidine side chain can act as an effective nucleophile, as an electrophile and can participate both as donor and acceptor in hydrogen bonds [25].

In addition to these interactions, histidine can coordinate a wide variety of divalent transition metal ions (Zn, Ni, Cu, Fe, Co, Mn) via one or both of the deprotonated N atoms (Figure 1B) [27]. This is pH dependent since the nitrogen needs to be deprotonated (i.e., histidine is unable to bind metal under acidic conditions). Furthermore, the coordination number and geometry of the metal complex is highly dependent on which metal ion is being coordinated. For example, Zn^2+^ tends to prefer a tetrahedral geometry, while Ni^2+^ prefers square pyramidal or octahedral [19,20,27,28,29]. These different configurations have very important implications for the functional role of these complexes in both catalytic and mechanical contexts. Histidine–metal coordination is a common motif observed in enzymes playing catalytic roles; however, relevant to the topic of this review, His–metal bonds are also found as mechanical load-bearing bonds, where they contribute to material properties such as hardness, stiffness, toughness and most importantly, self-healing capacity [4,11].

The first observation and clear description of a mechanical role for His–metal interactions was in the hard biting parts of marine worms and arthropods [30,31,32,33]. For example, ragworms (*Nereis virens*), commonly used as bait for fishing, are marine polychaetes that live beneath the sediment in intertidal mud flats and possess a pair of hard and stiff mandibles used for crushing and macerating prey [34]. The so-called jaws are comprised up to 90 wt % of protein depending on the region of the jaw and up to 8 wt % Zn^2+^ [30,34]. Biochemical analysis has revealed that the main structural component is a His- and Gly-rich protein, comprised of 27 mol % histidine [35]. Spectroscopic investigation has revealed that Zn^2+^ is coordinated on average by three histidine residues [31] and mechanical analysis has revealed that when metals are chemically removed, the stiffness and hardness of the jaws are reduced by over 65% [34]. However, properties are fully recovered upon incubation with Zn^2+^ ions, highlighting the inherently reversible nature of these supramolecular interactions [30,34]. Similar to *Nereis virens*, worms from the species *Glycera dibranchiata* utilize histidine–metal interactions (in this case His–Cu^2+^ complexes) to mechanically stabilize the jaws. Likewise, His–metal interactions have been shown to enhance mechanical properties of spider fangs and insect mandibles to varying degrees, indicating that this strategy has evolved independently in a number of different biological systems [11,32,33]. In all these examples, the role of His–metal coordination is related to a stiffening or hardening of biting parts. However, the role His–Zn^2+^ interactions in the mussel byssus is much different and in contrast to biting parts, is related to dissipative properties such as toughness, hysteresis, and most importantly for the focus of this review, self-healing capacity.

## 2. Mussel Byssus as Inspiration for Self-Healing Polymeric Materials

### 2.1. Mussel Byssus Background

**Figure 2:**
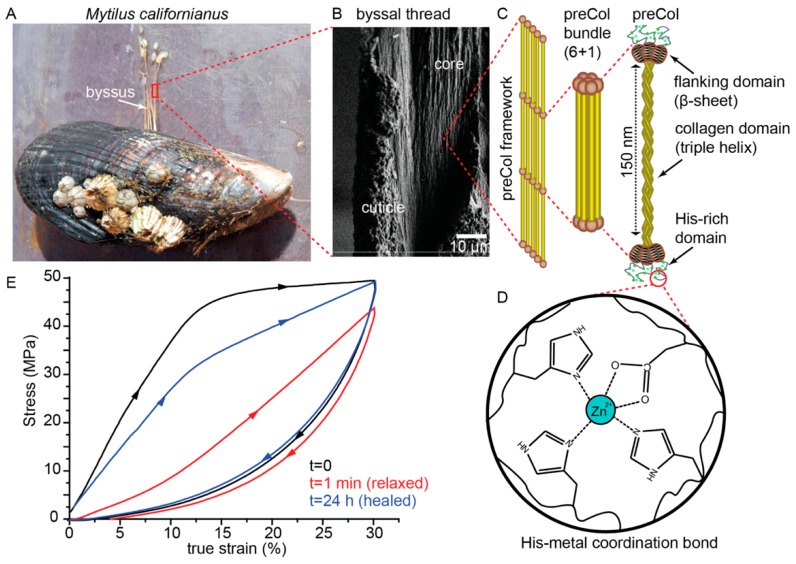
Mussel byssus healing and hierarchy. (**A**) Mussels from the genus *Mytilus* utilize proteinaceous attachment fibers collectively called a byssus to attach to hard surfaces on rocky seashores. (**B**) The distal thread core (surrounded by a thin protective coating) provides the mechanical resistance to crashing waves. (**C**) The thread core consists of multidomain collagenous proteins known as prepepsinized collagens (preCols) which are hierarchically organized into a semi-crystalline framework. (**D**) The histidine-rich domains (HRDs) at the ends of preCols are known to coordinate Zn^2+^ ions, which function to influence mechanical performance. (**E**) During cyclic tensile testing, threads exhibit a large mechanical hysteresis, but undergo damage when loaded past the yield point. Autonomous and intrinsic self-healing occurs over time to recover toward initial properties. Reprinted from [4], Copyright 2016, with permission from Elsevier.

Many marine mussel species, most notably *Mytilus* spp., attach to hard substrates in the rocky intertidal zone of wave-swept seashores by using a collection of protein-based attachment threads, known as a byssus (Figure 2) [36]. The individual byssal threads comprising a byssus (typically 20–50 threads) are secreted one by one in a formation process reminiscent of polymer injection molding [37]. At the end in contact with the substrate, a byssal thread terminates in a foamy underwater adhesive known as the plaque. At the end in contact with the mussel, the stem of the byssus is anchored into the soft tissue [36]. In between the plaque and stem is the thread itself, which is further divided into two mechanically, morphologically, and compositionally distinct parts known as the distal and proximal thread [38]. The distal region has a fibrous appearance, whereas the proximal region is much wider and folded with a wrinkled appearance. Surrounding the fibrous inner core of both the distal and proximal thread is a 2–5 µm thick protective coating, known as the cuticle [39,40]. In the current review, we focus on the distal thread core as this is the region responsible for the self-healing behavior observed in byssal threads (Figure 2A,B).

Mechanical stress–strain curves of whole mussel threads reveal that the mechanical behavior of byssal threads is dominated by the deformation response of the distal region of the thread because it is much stiffer than the proximal region and because it makes up more than two-thirds of the length of thread [41]. Byssal threads, like most proteinaceous biological fibers, are viscoelastic and thus, the mechanical response is highly dependent on loading rate [4,42]. However, in the typical loading regimes tested, the distal byssal thread displays a very characteristic deformation behavior, consisting of an initially stiff elastic region, followed by a well-defined yield point occurring between 10% and 15% true strain (Figure 2E). Following the yield point, there is an elongated and essentially flat yield plateau until about 40% strain, after which point there is a pronounced strain stiffening until more than 100% strain where mechanical failure occurs [41]. The actual material property values vary depending not only on loading rate, but also on mussel species and abiotic features such as seasonal variation, nutrition, water chemistry, and more [43,44,45,46], and thus different studies report a wide range of values [41,42,47,48]. However, stiffness values of up to 1 GPa have been reported for the California mussel (*Mytilus californianus*), whereas strain energy to break (i.e., toughness) values have been reported as high as 45 MJ/m^3^, comparable to that of polyaramid fibers, such as Kevlar, which are used in ballistic panels, making the byssus, along with spider dragline silk, one of the toughest biopolymeric fibers reported [47].

During cyclic tensile loading of threads to strain values below the ultimate strain, byssal threads exhibit hysteresis values of up to 70% (Figure 2E), indicating a remarkable propensity for dissipating mechanical energy, consistent with their assumed function in damping the impact of crashing waves on mussel beds [42]. However, if a thread is cycled twice in quick succession, it becomes apparent during the second cycle that lasting damage has occurred, evidenced by a drastic drop in stiffness and hysteresis to ≈30% of native values [42,49,50]. This is not unusual in a polymeric material taken past its yield point as it typically indicates plastic damage [49]. However, what is unusual and highly remarkable is that, given time to rest, damaged byssal threads recover toward initial material properties in a time- and temperature-dependent self-healing process, which is both autonomous and intrinsic (Figure 2E) [42,49,50]. This is even more impressive considering that the thread is acellular and comprised entirely of proteins.

While it has been reasonably proposed that the cuticle might contribute to this tensile behavior of the distal threads, since it surrounds the thread, it comprises only a small volume (>10%) of the total thread and furthermore, it was shown that thread sections with the cuticle removed by laser dissection exhibit a load-displacement curve nearly identical to a native distal thread [4]. Thus, it was proposed that the inner fibrous core of the distal byssal thread is the primary source of high toughness, high hysteresis, and the self-healing response. Therefore, to understand the chemical level source of this behavior, one must consider the composition of the core.

Early ultrastructural, histological, and X-ray diffraction studies of the distal region of byssal threads suggested the presence of a collagenous protein in the core [51,52]; however, detailed biochemical analysis of the core was initially hampered due to the heavily cross-linked nature of the proteins within. In 1994, Qin and Waite [53] were able to extract three distinct collagenous fractions of the core proteins by prolongated pepsin digestion of the thread, which they name prepepsinized collagens or preCols for short (Figure 2C). The full putative sequences of the preCols were subsequently deduced from complementary DNA (cDNA) library sequencing, revealing that these mussel collagens are highly distinct from typical animal fibrillar collagen (e.g., type I collagen). Indeed, the most striking feature was the presence of repetitive noncollagenous domains on either side of the collagen domain, which were designated as the flanking domains and which resembled different known functional protein domains [54,55,56]. The flanking domains of the three preCol variants—preCol-D (distal), preCol-NG (non-graded), and preCol-P (proximal)—resemble β-sheet forming poly-alanine domains from dragline silk, glycine-rich extensible sequences from flagelliform silk and proline, and glycine-rich sequences from elastin, respectively. Notably, while preCol-NG is evenly distributed throughout the thread, preCol-P is found only in the proximal end of the thread, whereas preCol-D is found in the distal region [57,58].

Although shorter and initially considered less remarkable than the flanking domains, the N- and C-terminal domains of all preCols consist of 20–100 amino acid stretches that are enriched in histidine to about 20 mol % [58]. However, recent findings reveal that these short domains, given the name histidine-rich domains (HRDs), play a crucial role in the high toughness and self-healing capacity of byssal threads (Figure 2C) [50,59]. Indeed, shortly after the discovery of the HRDs, it was proposed by Waite et al. [58] that the histidine might interact with high content of transition metal ions (Zn^2+^, Cu^2+^, Ni^2+^) known to be present in threads (Figure 2D). The first clear evidence for the potential role of metal coordination in the byssus were chelation experiments using ethylenediaminetetraacetic acid (EDTA) in which metal ions were removed from threads, resulting in a drastic loss of stiffness and perturbation of self-healing capacity [49]. While these experiments implicated the metal ions in the mechanical performance, they did not point a finger squarely at histidine. Based on the high sensitivity of histidine protonation (p*K*_a_ 6.5) to pH changes, researchers investigated the effect of incubating threads in solutions of different pH on mechanical performance [60]. It was found that the stiffness could be decreased by 50% when incubated below pH 5.0 compared to seawater pH ≈ 8.0 and that the stiffness decreased as a function of pH in a sigmoidal fashion with a halfway point around the p*K*_a_ of histidine. Furthermore, it was found that treatment at pH 4.0 resulted in a complete loss of the ability to heal [36,50].

To gain further evidence for the role of His–metal interactions, X-ray absorption spectroscopy (XAS) was performed at the Zn Kα-edge in order to investigate the first and second shell coordination environment in Zn^2+^ ions in native distal threads [59]. X-ray absorption near edge structure (XANES) analysis indicated a likely first shell coordination number of 5, while extended X-ray absorption fine structure (EXAFS) analysis suggested that these five ligands originated on average from three histidine residues (each providing coordination by one nitrogen atom) and one aspartate residue (providing coordination by two oxygen atoms) (Figure 2D). Moreover, EXAFS analysis of Zn Kα-edge spectra from stretched and damaged threads revealed significant changes in the Zn^2+^ coordination structure including exchange of amino acid ligands with water and increase of coordination bond lengths. Healed threads, however, showed a shift back toward a native coordination structure. Thus, the results from XAS strongly support a model of byssus thread self-healing in which His–metal interactions, presumably present in the HRDs, act as reversible sacrificial bonds [59]. This is consistent with previous atomic force microscopy (AFM)-based investigations utilizing single-molecule force spectroscopy (SMFS), which showed that histidine–metal interactions are strong, yet reversible with breaking forces between 22 and 60 pN at 0.5 µm/s loading rate [22]. Moreover, synthetic peptides with HRD sequences were conjugated to soft polyethylene glycol (PEG) colloidal particles, enabling colloidal probe spectroscopy between identical layers of HRD peptides, which showed a metal ion dependent increase in interaction energy [61]. These in vitro investigations add further weight to a model of byssal thread healing that is reliant on histidine–metal ion interactions.

### 2.2. Influence of Hierarchical Structure on Byssus Self-Healing

**Figure 3:**
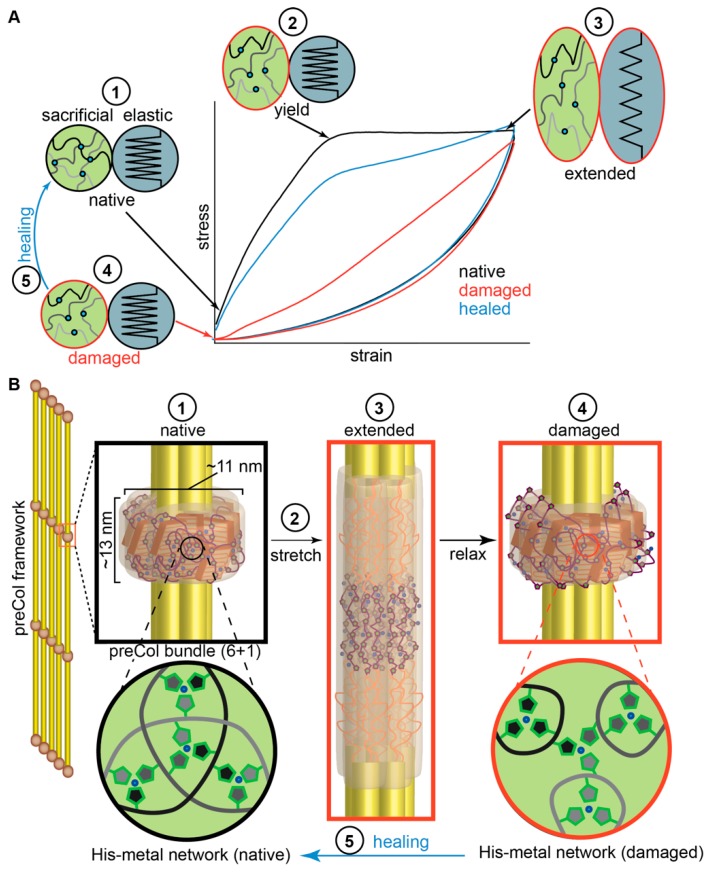
Model of byssal thread damage and self-healing. (**A**) Schematically, the byssus healing mechanism can be illustrated as a combination of a sacrificial bonding network and a source of elastic hidden length. The sacrificial network (1) begins to rupture at the yield point (2), transferring load to the elastic hidden length causing it to extend (3). When load is removed, the elastic component drives recovery of the initial length, but the sacrificial network is damaged (4). However, over time, the sacrificial network recovers toward its native state (5). (**B**) Within the hierarchical semicrystalline structure of the distal byssal thread core, the sacrificial bond network is comprised of an optimal network of intermolecular His–metal bonds that surrounds cross β-sheet structure in the flanking domains, which serve as a source reversible hidden length. Reprinted from [4], Copyright 2016, with permission from Elsevier.

While His–metal interactions are clearly implicated as a main driver of the healing process, X-ray diffraction-based investigations provided further details of the critical role of higher order hierarchical structure in guiding this process (Figure 3). For example, the performance of HRD peptides is not able to fully explain the exceptional stiffness and toughness of the native threads determined by metal coordination and is not able to explain the time-dependence of healing since in peptide-based studies, recovery was essentially instant [59,61]. Wide-angle X-ray diffraction and small-angle X-ray scattering (WAXD and SAXS) studies of mussel byssal threads, as well as spectroscopic investigations provided deeper insights into these questions [4,50,62,63,64]. Analysis of byssal threads by WAXD data revealed a diffraction pattern dominated by peaks consistent with collagen triple helical structure, which is not surprising considering that the central collagen domain of preCols comprises about 50% of the total preCol sequence [50]. However, a notable peak corresponding to the presence of cross-β-sheet structure was also evident, likely originating from the preCol-D flanking domains [4], which was also supported by nuclear magnetic resonance (NMR) and Fourier-transform infrared (FTIR) spectroscopic studies [62,63]. SAXS analysis, on the other hand, provided information about the higher order organization of the preCols into 6+1 hexagonal bundles that are further arranged laterally in quasi hexagonal arrays with center to center spacing of ≈11 nm [64] (Figure 2C and Figure 3B), which is consistent with earlier AFM-based studies [65]. Axially, the preCol bundles are arranged in a semicrystalline fashion with a clear stagger of ≈13 nm, which is remarkable considering that byssal threads are formed in just minutes as a secretion of soluble protein precursors under ambient conditions [37]. Within this structure, it was proposed that the flanking domain β-sheets are essentially embedded in a highly dense metal cross-link network created via interactions between the HRDs from neighboring preCol bundles (Figure 3B) [4].

These findings highlight the complex hierarchical organization of the preCols at length scales from the atomic scale up to the micron scale. Hierarchical structure is a nearly omnipresent feature in biological materials and is often associated with improved mechanical performance [8,66], particularly increased toughness, and possibly, in this case, self-healing. To examine this hypothesis, researchers coupled WAXD and SAXS measurements with in situ mechanical testing, observing the real time changes in multiscale protein structure as a function of mechanical loading. These combined results provided clear evidence that important characteristic features of distal byssal threads (e.g., high toughness and self-healing) originate from a synergistic response of the flanking domains and HRDs to applied forces (Figure 3) [64,67], while the collagen domain exhibit a lower deformation at higher strains [50]. More specifically, the current model proposes that the HRD–metal bonds act as a sacrificial network that resists deformation up to ≈10–15% strain contributing to material stiffness [59]. However, at a critical force indicated by the yield point, the His–metal bonds in the HRD network begin to rupture, transferring the applied load to the β-sheets of the flanking domains, leading them to unfold, which provides hidden length that contributes to the macroscopic extensibility of the thread (Figure 3) [4,63]. Results indicated that HRD metal cross-link networks from individual bundles ruptured consecutively at an essentially constant force during the yield plateau [4,64]. When load was removed from the thread, it was observed that the β-sheets refold on very short time scales, but that the metal coordination network did not return immediately to its native form (i.e., bond lengths are longer). Over time, the His–Zn complexes return back toward a native-like configuration, likely via bond exchange, which correlates with the healing process [59]. Indeed, investigation of the effect of incubation temperature on the observed healing rate has suggested an activation energy for the healing process of ≈0.9 eV (87 kJ/mol or 21 kCal/mol), which is consistent with the breaking and re-forming of metal coordination bonds [50,59]. Thus, byssal thread self-healing appear to involve a process of dynamic bond exchange until a thermodynamically stable, kinetically trapped His–metal bond network is achieved, which is strongly related to the hierarchical structure of the thread.

## 3. Bioinspired Metallopolymers

### 3.1. General Aspects of Metal–Ligand-Based Polymers

After their discovery, synthetic chemists realized that metal coordination complexes have appealing properties with regard to optical behavior, electronic properties, and more recently, mechanical performance when combined with polymeric backbones [68,69,70]. Indeed, many different metal–ligand interactions have been implemented into synthetic polymers as versatile building blocks [68]. These materials, the so-called metallopolymers, are a subclass of the rich family of supramolecular polymers. Metallopolymers combine both structural elements (i.e., metal complexes and polymers) within a single material. Consequently, they also feature the corresponding properties derived from both subunits. For example, the metal complexes can contribute to properties such as mechanical reversibility [71,72], absorption/emission in the visible spectrum [70,73], and electronic properties [71], while the polymer chains contribute to the processability, the thermal properties (i.e., glass transition or melting temperature) and the tunability of the polarities of the overall assemblies [68]. The interplay of metal complex as well as polymeric material can be utilized to design tailor-made materials, which are interesting candidates for a variety of different applications [69,74]. Thus, metallopolymers have been utilized as emitters in organic light emitting diodes [75,76], in biomedical applications [77] (e.g., as anticancer drugs [78] or as imaging agents [79]), as shape-memory polymers [80] and, finally, as self-healing materials [81]. In particular, the latter approach is based on the reversibility of corresponding metal–ligand interactions and the flow behavior of the polymers. Therefore, these materials feature the ability to restore their mechanical properties after damage. The first examples of synthetic healable metal–ligand-based systems focused on non-natural ligand systems (e.g., terpyridine [82] and 2,6-*bis*-(1′-methyl-benzimidazolyl)pyridine [83]). Those structural moieties were chosen due to their well-investigated properties in polymer science and their tunable supramolecular binding strength (by the choice of the metal ion). The investigations regarding self-healing have revealed that these materials feature the ability for crack healing. In contrast to biological systems, an external stimulus is still required in order to activate the reversibility of the metal–ligand interactions as well as the flow behavior of the polymer. For this purpose, light [83] or heat [82] have been utilized. Furthermore, an additional study revealed a strong dependency of the healing ability from the chosen metal salt [84]. Thus, very weak metal–ligand interactions resulted in an efficient crack healing (i.e., in short times and at comparatively low temperatures). Furthermore, the counter ion influences the healing ability since it can reduce the binding strength of the supramolecular interaction [85]. Nevertheless, these synthetic materials do not reach the efficiency/ability known from nature in mussel byssus threads, since an external stimulus is still required in order to enable healing. The autonomous self-healing behavior can be reached by other self-healing approaches, which are, e.g., based on an external healing agent encapsulated into a polymeric matrix [6]. However, those approaches do not utilize the naturally occurring dynamic metal–ligand interactions, since these self-healing materials rely on the encapsulation of liquid healing agents, which are released upon damage [86].

### 3.2. His-Functionalized Polymers: Using Imidazole plus Metal Ions to Achieve Dynamic and Self-Healing Ability

Among the initial efforts to translate extracted principles from the mussel byssus into synthetic materials [87,88], two main approaches can be distinguished. In the first approach, the interaction between DOPA via its catechol side chain and metal ions was utilized for the synthesis of artificial mussel-inspired metallopolymers [24,89]. These reports mainly deal with applications as coatings [90], adhesives [91] as well as self-healing materials [89,92,93]. Iron(III) was mainly utilized as a metal ion for a sufficient binding to the catechol groups [89,94,95]; however, other metals (or semi-metals) like boron, aluminum, vanadium, or gallium have also been utilized [95,96,97,98,99]. As already mentioned, these efforts are well-reviewed elsewhere [24,90].

However, this review focuses specifically on the second approach, which is based on the interaction of histidine with different metal ions as described in Section 2. The amino acid histidine consists of an imidazole unit as well as an amine and a carboxylic-acid functionality. Within natural materials, the two latter functionalities form the amide bond in peptides, and only the imidazole can contribute to the binding of different metal ions. For the design of mussel-inspired materials, two approaches can be distinguished. Whereas the first one just focuses on the utilization of the imidazole (i.e., the main binding motif), the second approach uses the complete histidine moiety with the additional amine and carboxylate moieties. The latter approach results in a stronger metal–ligand bond due to the additional possible interactions of the amine with the corresponding metal ions [100]. Additionally, the metal–ligand binding strength can also be influenced using additional substituents (like protecting groups) at the imidazole moiety (e.g., the trityl group) [101]. Due to the steric demand as well as the blocking of a potential binding position, the supramolecular interaction is weakened by at least one order of magnitude.

Synthetic histidine-containing metallopolymers have been mainly utilized in two forms: gels or bulk materials (particularly films). Mostly, zinc(II) [102] and nickel(II) [103] have been utilized for the complexation. In contrast, copper(II) [104] and cobalt(II/III) [105] have been utilized less frequently. The applied polymer backbones range from very hydrophilic ones (e.g., peptides and poly(ethylene glycol) [106]) to very hydrophobic ones (e.g., poly(alkyl methacrylates) [102]). Consequently, the properties of the resulting metallopolymer can be tuned over a broad range and are mainly influenced by the choice of the ligand (histidine or imidazole), the metal ion, the polymer type as well as the fabrication (gel [107] or pure bulk material [102]). The large variety investigated in recent years is summarized in Table 2.

In the following, selected examples will be presented showing the broad range of different design principles engineered into histidine-based synthetic materials, as well as the different approaches to mimic nature in artificial materials. The first study using histidine–metal interactions in artificial materials by the group of Messersmith harnessed a relatively simple synthesis route to produce polymer gels, which enabled a detailed analysis of the supramolecular binding between the ligand and different metal ions, as well as the mechanical consequences of these interactions [110]. Specifically, tetra-arm PEG was prepared featuring histidine moieties as functional end group at each of the four arms [110]. After addition of metal salts (i.e., zinc(II), nickel(II), copper(II), and cobalt(II)), gel formation was observed, which could be correlated with the formation of histidine–metal complexes. Notably it was discovered that the free amine group was highly important for gel formation. If no amine group was present, gel formation was not observed. Rheological measurements of the gels were performed revealing the critical role of the specific metal ions in tuning the response via the supramolecular bonds. Later studies by Holten-Andersen’s group on a related polymer found that the relaxation time(s) and viscoelastic response of such hydrogels could be fine-tuned by combining different amounts of several different metal ions into the gels, based on their different supramolecular bond lifetimes [23].

Further investigations aimed to incorporate the histidine– and imidazole–metal interactions into synthetic polymers for the design of healable polymers, taking advantage of the dynamic reversibility of the interactions. A first approach was presented by the Guan group using the weak imidazole–zinc(II) bond [114]. The weak cross-linking resulted in excellent healing properties within minutes at room temperature. Furthermore, the mechanical properties could also be improved due to the utilization of a phase-separating graft copolymer containing both polystyrene as well as poly(acrylate). This phase separation also led to metal complex-rich domains as well as regions without any supramolecular junction. Such phase separation is also known from mussel byssus threads [118]. Thus, this approach revealed a system with a high comparability to the natural one showing also the potential of mussel-inspired polymers for self-healing applications.

**Figure 4:**
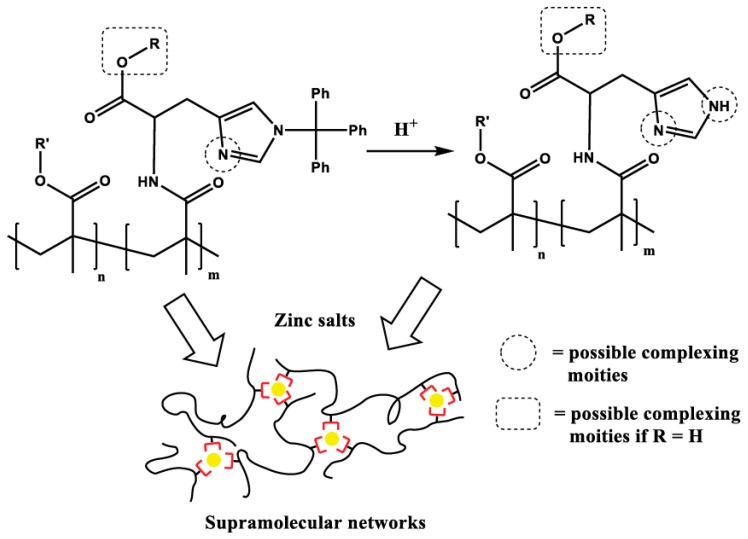
Schematic representation of the synthesis of supramolecular networks based on the interaction of zinc(II) with (protected) histidine-containing polymers (R = butyl; R’ = butyl or lauryl). Reprinted from [102], Copyright 2015, with permission from Elsevier.

**Figure 5:**
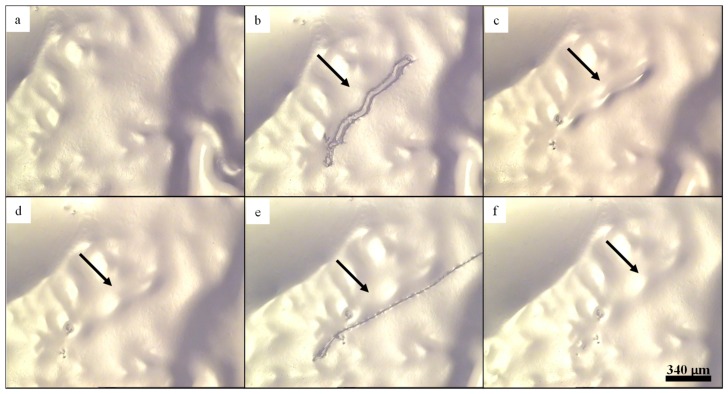
Healing of histidine-containing polymers. (**a**) Into a film of the histidine-based supramolecular polymer network, (**b**) a scratch was introduced and (**c**) healed for 40 h at 100 °C. (**d**–**f**) This procedure was repeated once with healing for 19 h at 100 °C. Reprinted from [102], Copyright 2015, with permission from Elsevier.

The incorporation of histidine moieties into synthetic polymers/polymer networks can be a quite challenging task. Due to the presence of different reactive functionalities of the histidine (i.e., –COOH, –NH_2_, imidazole) the utilization of protecting groups might be required. Thus, Enke et al. [102] presented an approach of designing a methacrylate-based histidine monomer featuring a trityl group at the imidazole unit in order to enable a sufficient functionalization. The protecting group could also be deprotected after polymerization resulting in histidine-containing copolymers (Figure 4). Furthermore, the binding strength of those moieties, with and without protecting groups revealed that the “pure” histidine featured a much stronger binding constant determined by isothermal titration calorimetry (ITC) compared to the protected one [101]. Subsequently, the ITC results were utilized for the preparation of histidine–zinc(II)-based supramolecular networks, which were processed into a polymer film. The healing ability of this film was studied in detail (Figure 5). In this case, the healing of scratches could be revealed at relatively low temperatures of about 40 to 100 °C. The extent of the healing depended on the structure of the chosen histidine (with or without protecting group), on the comonomer (lauryl or butyl methacrylate) as well as on the chosen zinc(II) salt (acetate, chloride, or nitrate). The presented approach featured a high structural comparability to nature due to the utilization of histidine instead of imidazole; however, the processing to films (instead of threads) as well as the lack of hierarchical structure is still a significant difference from the natural system.

Bioinspired design is a continuous feedback loop in which synthetic efforts inform biologists, just as much as nature informs polymer chemists. With this in mind, researchers utilized peptides based on small regions of the native preCol HRD sequences to understand its influence on the assembly and function of the natural system toward the design of new artificial materials. In the first study, the HRD peptides were utilized for the analysis of the binding behavior to metal ions showing the functional importance of the histidine-rich sequence analyzed by Raman spectroscopy and soft-colloidal probe force measurements [61]. It was further discovered that the HRD peptides undergo a rapid transition from unstructured to amyloid like β-crystalline structure when exposed to conditions mimicking the natural assembly process (i.e., increase from acidic to neutral pH) [119]. In a subsequent study, HRD peptides were covalently attached to a tetra-arm PEG polymer [28]. The starPEG–HRD polymer–peptide hybrid molecules were highly soluble at low pH, but could be induced to rapidly form hydrogels at neutral to basic pH even in the absence of metal ions, mimicking the natural byssus assembly process [28,37]. This stimuli-responsive behavior was tied to the formation of β-crystallite clusters between the peptides. Zinc(II) ions could be incorporated into the peptide clusters via histidine complexation; however, surprisingly there was not a pronounced effect on the gel storage modulus compared to gels without metals, but rather only an increase in damping behavior was observed. Notably, metal complexation by nickel(II), which is not typically found in native threads, resulted in a loss of β-crystalline structure and an order of magnitude drop in storage modulus [28]. Thus, it is clear that there is more to be discovered in terms of the intimate interaction between these metal-binding HRD domains and metal ions with regards to self-healing behavior.

### 3.3. Using Histidine–Metal Complexes for Advanced Healing Systems

The histidine–metal coordination can also be applied for advanced healing strategies. Within this context, two examples will be presented showing that this type of metal–ligand interaction can not only be utilized to transfer the knowledge learned from nature to artificial materials, but also be applied for either the understanding of the healing phenomenon of the supramolecular polymers [108] or for the design of healable sensors with tunable piezoresistivity [117]. The latter approach was realized by connecting the histidine moiety to cellulose nanocrystals (CNC). Afterward, this material was mixed with carbon nanotubes (CNTs) as well as epoxidized natural rubber (ENR) decorated with zinc(II) chloride. The obtained composite material was processed to form films and bulk materials. These materials revealed scratch and bulk healing ability. Furthermore, the electric conductivity of the composite materials could also be healed, which is a precondition for the application as sensor material. The sensor material was applied to detect human motion and could distinguish between various types of activities (pronunciation, coughing, and deep breathing) [117].

**Figure 6:**
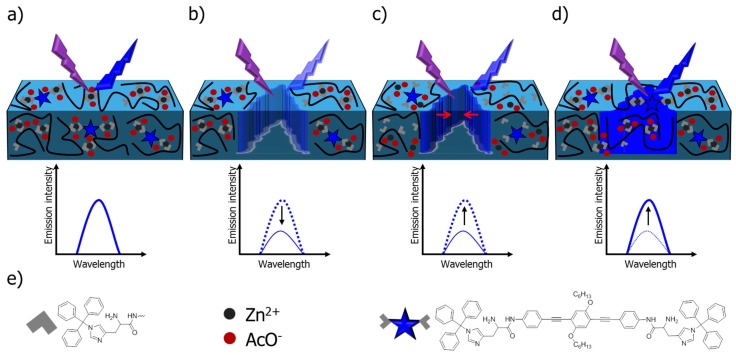
Schematic representation of the utilization of the emission of a sensor molecule (bottom right) for the monitoring of the healing process of supramolecular polymers. (**a**) The original material features a certain emission of light, (**b**) which is destroyed by a damage. (**c**) During the healing process, (**d**) the emission can be recovered until a complete healing is obtained. Reprinted with permission from [108]. Copyright 2018 American Chemical Society.

The other approach aimed to extract an understanding of the healing behavior, as well as monitoring of the process of healing [108]. For this purpose, a sensor molecule was designed featuring histidine capable of metal complexation, as well as a conjugated oligomer determining the optical properties (emission behavior). After processing of a film, a scratch was introduced resulting in a loss of emission behavior, which was monitored using confocal laser scanning microscopy (CLSM) (Figure 6). Consequently, the regeneration of the material could be monitored by the recovery of the emission behavior. The whole process could also be monitored in a depth-dependent manner and moreover, the emission signal could also be utilized for the quantification of the healing process.

### 3.4. Future Developments of Synthetic Bioinspired Metallopolymers

**Figure 7:**
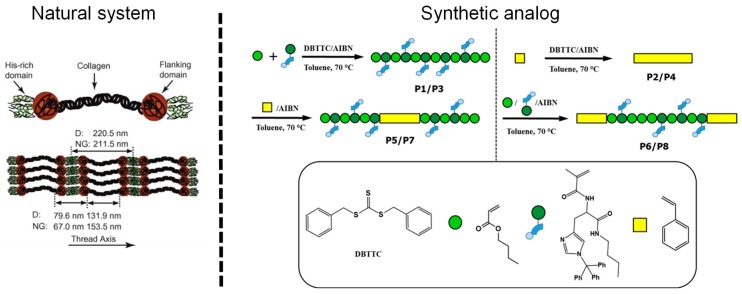
Schematic representation of the domain formation of the natural system (left) [50] and a first approach using block copolymers for the mimicking of that behavior in artificial materials (right) [120]. For this purpose, a block copolymer was synthesized featuring hard (polystyrene) and a soft (butyl acrylate with histidine ligand) domains. Afterward, these block copolymers were cross-linked by the addition of zinc salts resulting in metallopolymers with self-healing behavior. Reprinted from [50], Copyright 2009, with permission from Elsevier; and reproduced from [120] with permission from the Royal Society of Chemistry. AIBN: Azobisisobutyronitrile; DBTTC: *S*,*S*-dibenzyl trithiocarbonate; P1–8: Polymers.

The utilization of bioinspired histidine–metal interactions for the design of artificial materials is still in its infancy. The initial approaches described above primarily focused on the incorporation of histidine–metal complexes into synthetic materials, as well as some first studies regarding stimuli-responsive behavior and self-healing. However, the impressive behavior of the natural system, combining exceptional toughness with autonomous and intrinsic self-healing has not yet been reached. Future developments are required in order to bring synthetic materials closer to their natural archetypes. In particular, the complex structural hierarchy inherent in the preCol functional domains has not been successfully mimicked. Nevertheless, a first synthetic approach inspired by the multidomain structure of the preCols has been recently described by Enke et al. [120] utilizing block-copolymers with hard and soft segments. The soft segments featured histidine moieties, which were utilized for the design of supramolecular polymers capable for self-healing behavior (Figure 7).

Additionally, a principal feature of the natural system is the autonomous self-healing ability without the need for any another external stimulus, which is highly desirable from a biomimicry standpoint. The key to this property is thought to be the mechanical activation of the supramolecular bond occurring during deformation beyond the apparent yield point [121]. In other words, molecular-level damage of the metal complex network directly results in the subsequent activation of the healing process and, consequently, could be a potential avenue toward development of polymers exhibiting intrinsic and autonomous healing. Finally, the implementation of peptides into PEG showed that the role of the metal–ligand interaction and its specific influence on the mechanics and finally on the self-healing behavior is still not fully understood [28]. Indeed, it appears that the process is tightly linked to the higher order structure achieved by the regular backbone contortions of the protein chain [28]. Future research is required to further elucidate the multiscale details of this process from the molecular scale up through higher levels of hierarchy. Within this context, also the influence of the other amino acids available in natural systems must be investigated, in particular in artificial materials. A first approach was already presented showing that an additional aspartate unit in the polymer can improve the healing behavior while maintaining the mechanical performance [122]. Nevertheless, future developments are expected in this topic of research.

## 4. Conclusions

Based on natural selection occurring over eons, nature has evolved effective and efficient solutions to common engineering challenges also facing synthetic material design and implementation. In particular, biological organisms exhibit remarkable examples of intrinsic and autonomous self-healing in material structures that offer excellent archetypes for engineering of damage-tolerant synthetic polymers and composites of the future [3,4]. Several prominent examples, including the mussel byssus, harness metal coordination cross-links as strong, yet reversible sacrificial bonds that can contribute to material toughness and a capacity for self-healing [4,11]. Elucidation of the mechanisms underlying mussel byssus self-healing have revealed a critical role of histidine–metal coordination cross-links embedded in a complex hierarchical protein structure [4,59]. Initial efforts to replicate and explore the potential of imidazole and histidine functional groups in producing self-healing behavior in synthetic polymers are highly encouraging [23,102,110]; however, future advances will depend on more closely mimicking the natural hierarchical structure. Indeed, recent studies aimed at understanding the natural byssus assembly process are revealing important new insights that will be extremely useful to these continued efforts to mimic byssus healing [28,37,119].

## Figures and Tables

**Table 1:** Summary of self-healing phenomena found in natural systems.

	System	Proposed Mechanism	References
**Cellular**	Wound healing	Initial wound sealing by aggregation of platelets Cell migration to the damaged site, followed by new tissue formation Remodeling of acellular extracellular matrix	[7]
	Bone healing	Cells move to damage site and form a callus Cells produce new tissue (collagen fibers and spongy bone) Remodeling of spongy bone to compact bone Healing of microcracks occurs through constant resorption and formation of bone	[12]
	Latex-bearing plants	Sealing of injury by release and coagulation of fluid latex stored in microtubes Subsequent cell proliferation and growth processes	[13]
**Acellular**	Mussel byssus	Reversible sacrificial bonds and hidden length based on protein–metal coordination	[4]
	Caddisfly silk	Reversible inter- and intramolecular interactions between phosphoserine and Ca^2+^/Mg^2+^ ions	[14]
	Whelk egg capsules	Hidden length based on reversible unfolding and refolding of protein backbone (α-helix ⟷ β-sheet)	[15]

**Table 2:** Summary of the different utilized design strategies for the fabrication of histidine-containing synthetic polymers.

Authors	Ligand	Metal ion	Polymer	Bulk/Gel	Reference
Enke and colleagues	Histidine	Zinc(II)	Poly(methacrylate)	Bulk/Film	[101,102]
Ahner et al.	Histidine	Zinc(II)	Poly(methacrylate)	Bulk/Film	[108]
Tang et al.	Histidine	Nickel(II)	Polyacrylamide	Hydrogel	[109]
Fullenkamp et al.	Histidine	Zinc (II), copper(II), nickel(II), cobalt(II)	Poly(ethylene glycol)	Hydrogel	[110]
Grindy and colleagues	Histidine	Copper(II), nickel(II), cobalt(II)	Poly(ethylene glycol)	Hydrogel	[23,111,112]
Harrington and colleagues	Histidine	Zinc (II) and nickel (II)	Poly(ethylene glycol)/peptides	Hydrogel	[28,61]
Wegner et al.	Histidine	Cobalt(II/III)	Poly(ethylene glycol)	Hydrogel	[105]
Tang and colleagues	Histidine	Nickel(II)	Poly(ethylene glycol) and chitosan	Hydrogel	[106,113]
Pham et al.	Histidine	Nickel(II)	Peptide	Hydrogel	[103]
Mozhdehi and colleagues	Imidazole	Zinc(II), copper(II), cobalt(II)	Polystyrene-graft-poly(acrylate)	Bulk	[104,114]
Liu et al.	Imidazole	Zinc(II)	Poly(propylene glycol)	Bulk/Film	[115]
Xu et al.	Imidazole	Zinc(II)	Poly(acrylate)	Bulk/Film	[116]
Liu et al.	Imidazole	Zinc(II)	Cellulose	Bulk/Composite	[117]
